# Effects of empagliflozin on uric acid levels during acute heart failure recompensation: A sub‐analysis of the EMPAG‐HF trial (Effects of Empagliflozin on Diuresis and Renal Function in Patients with Acute Decompensated Heart Failure)

**DOI:** 10.1002/ejhf.3723

**Published:** 2025-07-09

**Authors:** Jurgen Bogoviku, Tien Dung Nguyen, Julian Georg Westphal, Franz Haertel, Pawel Aftanski, Sven Möbius‐Winkler, Martin Busch, P. Christian Schulze

**Affiliations:** ^1^ Division of Cardiology, Angiology and Intensive Medical Care, Department of Internal Medicine I University Hospital Jena Jena Germany; ^2^ Division of Nephrology, Department of Internal Medicine III University Hospital Jena Jena Germany

**Keywords:** Acute decompensated heart failure, Uric acid, Empagliflozin, SGLT2 inhibition

## Abstract

**Background:**

Sodium–glucose cotransporter 2 inhibitors improve prognosis in chronic heart failure as part of currently recommended therapeutic strategies. Elevated levels of serum uric acid (SUA) have been associated with worsening of outcomes in cardiovascular disease and may lead to hyperuricaemia and gout as well as exacerbation of renal failure. The effects of empagliflozin on SUA in patients with acute decompensated heart failure (ADHF) remain unknown.

**Methods and results:**

In the single‐centre, prospective, double‐blind, placebo‐controlled EMPAG‐HF trial, patients with ADHF were screened and randomized within 12 h following hospital admission to receive either empagliflozin or placebo in addition to standard medical treatment over 5 days. Sixty patients were enrolled and randomized irrespective of left ventricular ejection fraction or diabetes. Serum and urine uric acid were evaluated as part of the standard monitoring protocol. Baseline patient characteristics did not differ between the two groups. SUA increased in the placebo group during diuretic therapy but decreased in the empagliflozin group. At 3, 4 and 5 days, SUA was significantly lower in the empagliflozin group compared to placebo. The reduction in SUA following empagliflozin treatment was associated with an enhanced renal excretion of uric acid.

**Conclusions:**

Addition of empagliflozin to standard diuretic therapy may prevent the rise in SUA during decongestion in patients with ADHF and thus may prevent negative effects of hyperuricaemia including the occurrence of acute gout episodes.

## Introduction

Acute decompensated heart failure (ADHF) represents a clinically challenging aspect of heart failure (HF).^1^ Twenty‐six million patients live with HF^2^ with a prevalence of up to 10% among individuals over 70 years of age.^3^ The prognosis remains poor, despite modern therapeutic interventions; epidemiological data state an overall surviving of 80–90% at 1 year, 50–60% at 5 years and 30% at 10 years.^4^ Recurrent decompensation and subsequent episodes of acute HF in patients with chronic stable HF increases the risk of mortality.^5^ Acute HF decompensations are associated with the risk of an irreversible impairment of end‐organ function which itself carries prognostic impact. This is a pivotal aspect because of the impact on the management of HF, therapeutic interventions and prognosis.^6^

Elevated levels of serum uric acid (SUA) is a frequent condition in HF patients.^7^ An increase of 1 mg/dl in SUA is associated with an augmentation of all‐cause mortality by 4% and HF hospitalization by 28%.^8^ Furthermore, prior studies showed an impact of uric acid‐lowering drugs on markers of oxidative stress, endothelial function and peripheral blood flow in patients with chronic stable HF.^9^

Patients with acute HF receiving diuretic treatments may develop hyperuricaemia and acute renal failure with worsening of outcome.^10^ Its impact on kidney disease represents a pivotal aspect in HF patients since chronic kidney disease and worsening renal function are strong outcome predictors in HF.^11^, ^12^ Furthermore, the cardiorenal interaction may lead to challenging diuretic choices in hypervolaemia, and intensive diuretic strategies frequently require combination therapies.^13^ Consequently, clinicians often face a therapeutic dilemma in ADHF.

Sodium–glucose cotransporter 2 (SGLT2) inhibitors are part of the well‐established guidelines for medical therapy in HF and reduce mortality and hospitalizations in chronic HF patients.^14^, ^15^ SGLT2 inhibitors reduce SUA through enhancing fractional excretion of uric acid (FEUA).^16^ In the EMPAG‐HF (Effects of Empagliflozin on Diuresis and Renal Function in Patients with Acute Decompensated Heart Failure) trial, we have previously shown that empagliflozin improves the diuretic response in ADHF patients with volume overload without affecting renal function and serum electrolytes.^17^ In the present work, we performed a pre‐defined secondary analysis to assess changes in SUA in a prospective, randomized, placebo‐controlled setting.

## Methods

**Figure 1:**
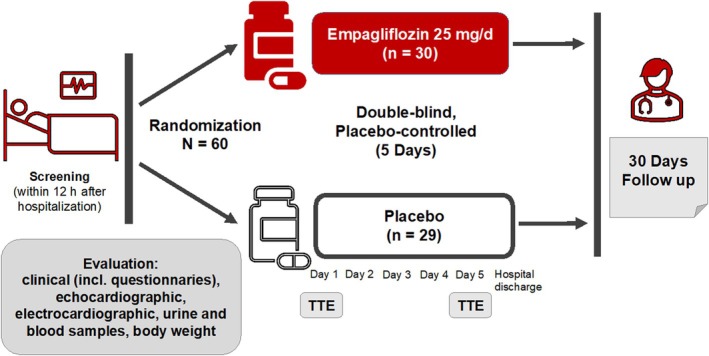
Study design of the EMPAG‐HF trial. TTE, transthoracic echocardiography.

The EMPAG‐HF (NCT04049045) trial was a prospective double‐blind, placebo‐controlled trial that tested the effects of SGLT2 inhibition with empagliflozin in addition to standard medical care compared to placebo on urine output as well as acute renal injury. Empagliflozin at a dose of 25 mg daily or placebo was administered within 12 h of hospital admission for 5 days in addition to standard medical care (*Figure* *1* and *Graphical Abstract*). The local Ethics Committee of the University Hospital Jena approved the protocol. All patients gave written informed consent. SUA levels were measured at baseline, then daily on the following 4 days, and at 30 days. Urine uric acid was assessed at 3 and 5 days as well as after hospital discharge at 30 days.

### Patient cohort

Patients who were admitted to our institution between June 2019 and April 2020 because of ADHF were screened for eligibility within 12 h. Sixty patients between 18 and 85 years with elevation of N‐terminal pro‐B‐type natriuretic peptide (NT‐proBNP) over 300 pg/ml were randomized. The aetiology of HF, ejection fraction, the presence of type 2 diabetes mellitus or impaired glucose tolerance were not relevant. Pregnancy for women of childbearing potential was evaluated through testing and adequate documentation of correct use of contraception. The exclusion criteria comprised the presence of type 1 diabetes mellitus, current treatment with SGLT2 inhibitors, intolerance or contraindication to furosemide or empagliflozin, chronic kidney disease with estimated glomerular filtration rate (eGFR) <30 ml/min/1.73 m^2^ or stage 2 acute kidney injury, acute HF without signs of congestion, indication for urgent angiography or administration of iodine‐based contrast agent within the next 6 days, unstable patients (inotropy/vasopressor or mechanical support dependent), planned operations, alcohol abuse (daily intake of >12 g in women and >24 g in men), or incapacity to understand the full extent of the study.

### Statistical analysis

All randomized patients were analysed and only patients without major protocol deviations took part of the per‐protocol evaluation. The complete description of sample size calculation, power, primary and secondary analysis has been previously published.^17^ We used two‐way mixed ANOVA to examine both between‐group and within‐group differences after checking for normality using the Shapiro–Wilk test. The significance level alpha is set to 0.05. Statistical tests were performed using R version 4.3.1.

## Results

### Demographics

The empagliflozin group comprised 30 patients and the placebo group comprised 29 patients. There were no differences in patient characteristics at baseline including left ventricular ejection fraction, NT‐proBNP, eGFR, and glycated haemoglobin (*Table* *1*).

**Table 1:** Baseline characteristics

Parameter	Empagliflozin (*n* = 30)	Placebo (*n* = 29)
Age, years	72.9 (68.8–77.1)	76.5 (73.4–79.7)
Female sex, *n* (%)	11 (36.7)	12 (41.4)
Body mass index, kg/m^2^	31.1 (27.5–34.7)	29.9 (27.1–32.6)
Heart rate, bpm	80 ± 17 (73–87)	79 ± 23 (70–88)
Systolic blood pressure, mmHg	139 ± 25 (129–148)	132 ± 21 (124–140)
Aetiology of heart failure, *n*/*N* (%)		
Ischaemic	5/17 (29.4)	5/16 (31.3)
Non‐ischaemic	8 (47.1)	5 (31.3)
History of cardiovascular disease, *n*/*N* (%)		
Atrial fibrillation	13/27 (48.1)	14/27 (51.9)
Type 2 diabetes mellitus	13/30 (43.4)	10/29 (34.5)
Hypertension	27/30 (90)	25/29 (86.2)
De novo heart failure, *n* (%)	18 (60)	14 (48)
Heart failure medication, *n* (%)		
Renin‐angiotensin system inhibitor	23 (76.7)	20 (69)
Sacubitril/valsartan	5 (16.7)	5 (17.2)
Mineralocorticoid receptor antagonist	7 (23.3)	4 (13.8)
Beta‐blocker	22 (73.3)	25 (86.2)
Previous treatment with loop diuretics	19 (63)	17 (59)
Previous treatment with thiazides	2 (6.7)	0
Laboratory parameters		
eGFR (ml/min)	58.2 ± 19.3 (51–66)	62.2 ± 18.2 (55–69)
Creatinine (mmol/L)	107 ± 29 (96–118)	98 ± 28 (87–109)
Bilirubin (μmol/L)	13.3 ± 9.4 (9.8–16.8)	16.8 ± 9.6 (13–20.6)
NT‐proBNP (pg/ml)	4726 ± 4516 (2939–6513)	4823 ± 4995 (2923–6724)
Serum uric acid (μmol/L)	458 ± 111 (411–505)	488 ± 151 (421–555)

Values are given as median (interquartile range), *n/N* (%), or mean ± standard deviation (interquartile range).

eGFR, estimated glomerular filtration rate; NT‐proBNP, N‐terminal pro‐B‐type natriuretic peptide.

### Serum parameters of renal function

**Figure 2:**
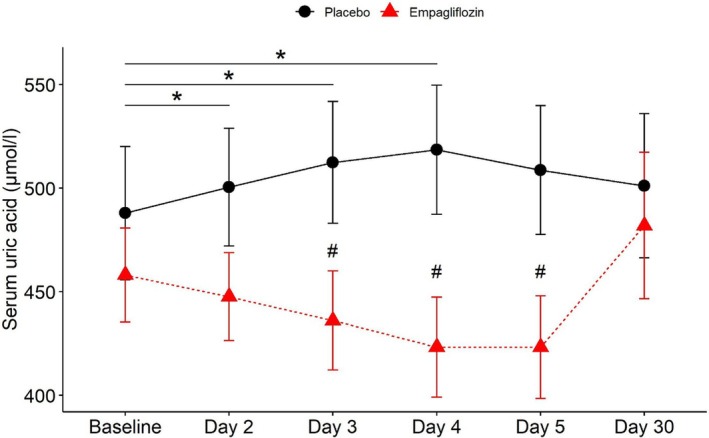
Changes in serum uric acid upon treatment with empagliflozin or standard diuretics. Data are presented as mean ± standard error of the mean. *Significantly different versus baseline (Day 2: *p* = 0.045, Day 3: *p* = 0.018, Day 4: *p* = 0.021). ^#^Significantly different versus placebo at the same time points (Day 3: *p* = 0.049, Day 4: *p* = 0.021, Day 5: *p* = 0.037).

In the placebo group, SUA levels increased and were higher than baseline (487.9 ± 32.21 μmol/L) at day 2 (500.4 ± 28.37 μmol/L), day 3 (512.4 ± 29.43 μmol/L) and day 4 (518.5 ± 31.14 μmol/L) reaching statistical significance already at day 2 (*Figure* *2*). By contrast, in the empagliflozin group, SUA levels tended to decrease compared to baseline (458 ± 22.71 μmol/L) and were significantly lower compared to placebo already at day 3 (436.1 ± 23.94 μmol/L, *p* = 0.049), furthermore at day 4 (423.2 ± 4.12 μmol/L) and day 5 (423.2 ± 24.75 μmol/L). After cessation of empagliflozin treatment on day 5, SUA levels in both groups at 30 days (placebo: 501.1 ± 34.75 μmol/L; empagliflozin: 481.9 ± 35.38 μmol/L) were similar and comparable to baseline values (*Figure* *2*). As previously published, the addition of empagliflozin (25 mg/day) to standard medical care in ADHF patients decreased cumulative dose of loop diuretics and increased diuretic efficiency compared to placebo.^17^

### Urine parameters of renal function

**Figure 3:**
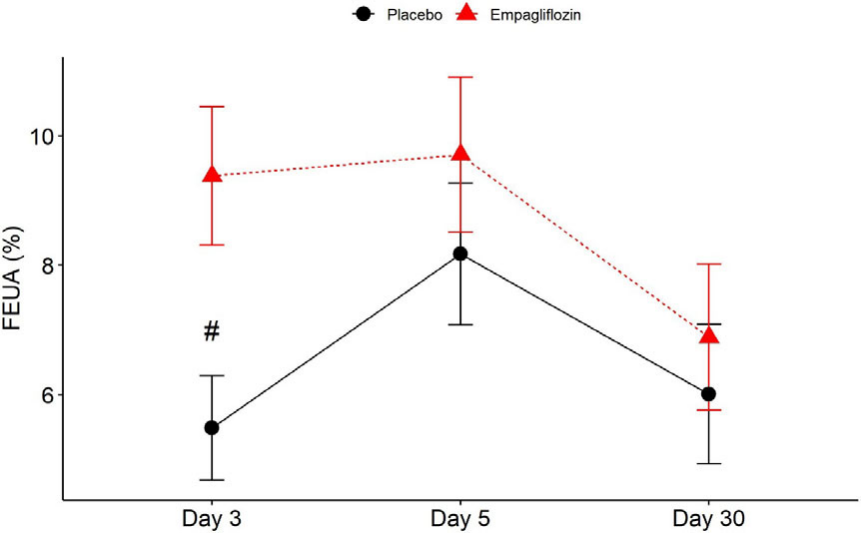
Fractional excretion of uric acid (FEUA) after 3, 5 and 30 days of treatment. Data are presented as mean ± standard error of the mean. ^#^Significantly different versus placebo (Day 3: *p* = 0.008).

The reduction in SUA in the empagliflozin group was associated with an early increase in the FEUA. At day 3, FEUA in the empagliflozin group was significantly higher compared to placebo (9.38 ± 1.07% vs. 5.49 ± 0.81%, *p* = 0.008). At day 5, while FEUA in the empagliflozin group (9.71 ± 1.2%) remained high, FEUA in controls (8.17 ± 1.1%) had also increased and the difference between groups was no longer significant (*Figure* *3*). At 30 days, FEUA decreased to baseline and was similar between the two groups (placebo: 6.01 ± 1.08%; empagliflozin: 6.89 ± 1.13%). We found no correlation between serum and urine uric acid levels both in the placebo and treatment groups, SUA levels and cumulative dose of diuretics in the treatment group and urine albumin and SUA on day 5 (online supplementary *Figures* *S1*–*S3*). Also, we did not detect any differences in SUA between *de novo* HF and ADHF (online supplementary *Figure* *S4*). Similarly, although female patients tended to have higher levels of SUA, the difference by treatment (*p* = 0.041) but not by gender (*p* = 0.051) was statistically significant.

## Discussion

This study shows for the first time in a randomized setting that empagliflozin reduces the accumulation of SUA under intensified diuretic therapy as part of decongestive measures in patients with ADHF. To the best of our knowledge, this represents a novel aspect, considering the enhancement of urine output without renal injury or relevant metabolic shifts under SGLT2 inhibition additive to standard diuretic therapy in ADHF patients.

Uric acid represents a prognostic relevant marker in HF and is associated with disease progression.^18^ Intensified diuretic regimes elevate SUA as a consequence of interference with renal uric acid excretion.^19^ Of note, many uric acid‐lowering therapies have failed to demonstrate a prognostic effect in various disease states including HF.^20^

Elevated levels of SUA lead to oxidative stress, endothelial injury and vasoconstriction mostly through mitochondrial injury, neurohumoral activation and reactive oxygen species production.^21^ Furthermore, the role of renal focal depositions of urate crystals with granulomatous reaction with accumulation of macrophages and T‐lymphocytes has been previously described.^22^ Many studies have also highlighted the correlation between elevated SUA levels and acute kidney injury including postoperative adaptation,^23^ sepsis^24^ and in hospitalized patients.^25^ Already 1 mg/dl increase in SUA leads to a 16% higher risk of acute kidney injury.^26^

The reduction of SUA through empagliflozin treatment in ADHF has several repercussions. SGLT2 inhibition reduces SUA in chronic HF patients and may contribute to the positive impact on prognosis.^8^, ^16^ In this study, we could demonstrate an early reduction of SUA already 24 h after treatment initiation and an increase after cessation (at 30‐day follow‐up). This early reduction in SUA might contribute to the stability of renal function despite enhancing urine output as seen in the EMPAG‐HF trial.^17^ Known pathophysiological effects of the SGLT2 inhibition,^27^, ^28^ which have been confirmed in large clinical studies^29^ represent the complexity of the effects of SGLT2 inhibition on renal function. The results of our current study imply that an acute reduction in SUA also contributes to the preservation of renal function. This, in our view, can be projected to all patients who present with ADHF regardless of left ventricular ejection fraction and diabetes status.

Here we demonstrate for the first time that a prognostically relevant drug such as the SGLT2 inhibitor empagliflozin is effective in the reduction of SUA during recompensation of patients with acute HF. Our data also confirm that intensive diuretic therapy leads to hyperuricaemia as described in previous studies,^30^ and that this side effect of intense diuretic therapy can be prevented by concomitant therapy with empagliflozin. Empagliflozin is safe and does not contribute to renal injury or relevant metabolic shifts, at the same time enhancing urine output in ADHF patients.^17^

Our study has several limitations. We enrolled 60 patients in a single‐centre, randomized trial. While the current analysis is a pre‐defined secondary analysis, the study lacks power for clinical outcome analysis including the frequency of gout episodes and the results are of exploratory nature. Furthermore, the investigated cohort comprises a specific patient group of ADHF patients with volume overload. Studies with a larger sample size that focus on the uricosuric effect of empagliflozin are necessary to verify our results.

Our data suggest that the additive treatment with empagliflozin in patients with ADHF may prevent the rise in SUA under intensive treatment with loop diuretics. This effect may be attributed to an improved renal elimination of uric acid. It remains to be clarified whether this uricosuric effect of empagliflozin also contributes to its prognostic benefits in HF and reduces clinical symptoms including the occurrences of hyperuricaemia and gout in the longtime.

## Supporting information


**Appendix S1.** Supporting Information.

## References

[1] Yancy CW , Jessup M , Bozkurt B , Butler J , Casey DE Jr , Colvin MM , *et al*. 2017 ACC/AHA/HFSA focused update of the 2013 ACCF/AHA guideline for the management of heart failure: A report of the American College of Cardiology/American Heart Association task Force on Clinical Practice Guidelines and the Heart Failure Society of America. Circulation 2017;136:e137‐e161. 10.1161/CIR.0000000000000509 28455343

[2] Ponikowski P , Anker SD , AlHabib KF , Cowie MR , Force TL , Hu S , *et al*. Heart failure: Preventing disease and death worldwide. ESC Heart Fail 2014;1:4–25. 10.1002/ehf2.12005 28834669

[3] Ponikowski P , Voors AA , Anker SD , Bueno H , Cleland JG , Coats AJ , *et al*. 2016 ESC Guidelines for the diagnosis and treatment of acute and chronic heart failure: The Task Force for the diagnosis and treatment of acute and chronic heart failure of the European Society of Cardiology (ESC). Developed with the special contribution of the Heart Failure Association (HFA) of the ESC. Eur J Heart Fail 2016;18:891–975. 10.1002/ejhf.592 27207191

[4] Jones NR , Hobbs FR , Taylor CJ . Prognosis following a diagnosis of heart failure and the role of primary care: A review of the literature. BJGP Open 2017;1:bjgpopen17X101013. 10.3399/bjgpopen17X101013 PMC6169931 30564675

[5] Lindmark K , Boman K , Stålhammar J , Olofsson M , Lahoz R , Studer R , *et al*. Recurrent heart failure hospitalizations increase the risk of cardiovascular and all‐cause mortality in patients with heart failure in Sweden: A real‐world study. ESC Heart Fail 2021;8:2144–2153. 10.1002/ehf2.13296 33751806 PMC8120394

[6] Heidenreich PA , Bozkurt B , Aguilar D , Allen LA , Byun JJ , Colvin MM , *et al*. 2022 AHA/ACC/HFSA Guideline for the management of heart failure: A report of the American College of Cardiology/American Heart Association Joint Committee on Clinical Practice Guidelines. J Am Coll Cardiol 2022;79:e263–e421. 10.1016/j.jacc.2021.12.012 35379503

[7] Borghi C , Palazzuoli A , Landolfo M , Cosentino E . Hyperuricemia: A novel old disorder‐relationship and potential mechanisms in heart failure. Heart Fail Rev 2020;25:43–51. 10.1007/s10741-019-09869-z 31745840

[8] Huang H , Huang B , Li Y , Huang Y , Li J , Yao H , *et al*. Uric acid and risk of heart failure: A systematic review and meta‐analysis. Eur J Heart Fail 2014;16:15–24. 10.1093/eurjhf/hft132 23933579

[9] Doehner W , Schoene N , Rauchhaus M , Leyva‐Leon F , Pavitt DV , Reaveley DA , *et al*. Effects of xanthine oxidase inhibition with allopurinol on endothelial function and peripheral blood flow in hyperuricemic patients with chronic heart failure: Results from 2 placebo‐controlled studies. Circulation 2002;105:2619–2624. 10.1161/01.cir.0000017502.58595.ed 12045167

[10] Palazzuoli A , Ruocco G , Pellegrini M , Beltrami M , Giordano N , Nuti R , *et al*. Prognostic significance of hyperuricemia in patients with acute heart failure. Am J Cardiol 2016;117:1616–1621. 10.1016/j.amjcard.2016.02.039 27040576

[11] Damman K , Valente MA , Voors AA , O'Connor CM , van Veldhuisen DJ , Hillege HL . Renal impairment, worsening renal function, and outcome in patients with heart failure: An updated meta‐analysis. Eur Heart J 2014;35:455–469. 10.1093/eurheartj/eht386 24164864

[12] Shirakabe A , Okazaki H , Matsushita M , Shibata Y , Goda H , Uchiyama S , *et al*. Hyperuricemia complicated with acute kidney injury is associated with adverse outcomes in patients with severely decompensated acute heart failure. Int J Cardiol Heart Vasc 2019;23:100345. 10.1016/j.ijcha.2019.03.005 31321285 PMC6612750

[13] Guo L , Fu B , Liu Y , Hao N , Ji Y , Yang H . Diuretic resistance in patients with kidney disease: Challenges and opportunities. Biomed Pharmacother 2023;157:114058. 10.1016/j.biopha.2022.114058 36473405

[14] Anker SD , Butler J , Filippatos G , Ferreira JP , Bocchi E , Böhm M , *et al*.; EMPEROR‐Preserved Trial Investigators . Empagliflozin in heart failure with a preserved ejection fraction. N Engl J Med 2021;385:1451–1461. 10.1056/NEJMoa2107038 34449189

[15] McMurray JJV , Solomon SD , Inzucchi SE , Køber L , Kosiborod MN , Martinez FA , *et al*.; DAPA‐HF Trial Committees and Investigators . Dapagliflozin in patients with heart failure and reduced ejection fraction. N Engl J Med 2019;381:1995–2008. 10.1056/NEJMoa1911303 31535829

[16] Iwata Y , Notsu S , Kawamura Y , Mitani W , Tamai S , Morimoto M , *et al*. The effect of dapagliflozin on uric acid excretion and serum uric acid level in advanced CKD. Sci Rep 2023;13:4849. 10.1038/s41598-023-32072-y 36964174 PMC10039024

[17] Schulze PC , Bogoviku J , Westphal J , Aftanski P , Haertel F , Grund S , *et al*. Effects of early empagliflozin initiation on diuresis and kidney function in patients with acute decompensated heart failure (EMPAG‐HF). Circulation 2022;146:289–298. 10.1161/CIRCULATIONAHA.122.059038 35766022

[18] Packer M . Uric acid is a biomarker of oxidative stress in the failing heart: Lessons learned from trials with allopurinol and SGLT2 inhibitors. J Card Fail 2020;26:977–984. 10.1016/j.cardfail.2020.08.015 32890737

[19] Schrijver G , Weinberger MH . Hydrochlorothiazide and spironolactone in hypertension. Clin Pharmacol Ther 1979;25:33–42. 10.1002/cpt197925133 363334

[20] Doehner W , Anker SD , Butler J , Zannad F , Filippatos G , Ferreira JP , *et al*. Uric acid and sodium‐glucose cotransporter‐2 inhibition with empagliflozin in heart failure with reduced ejection fraction: The EMPEROR‐Reduced trial. Eur Heart J 2022;43:3435–3446. 10.1093/eurheartj/ehac320 35788657 PMC9492270

[21] Sharaf El Din UAA , Salem MM , Abdulazim DO . Uric acid in the pathogenesis of metabolic, renal, and cardiovascular diseases: A review. J Adv Res 2017;8:537–548. 10.1016/j.jare.2016.11.004 28748119 PMC5512153

[22] Kim YG , Huang XR , Suga S , Mazzali M , Tang D , Metz C , *et al*. Involvement of macrophage migration inhibitory factor (MIF) in experimental uric acid nephropathy. Mol Med 2000;6:837–848. 10.1007/BF03401822 11126199 PMC1949919

[23] Ejaz AA , Alquadan KF , Dass B , Shimada M , Kanbay M , Johnson RJ . Effects of serum uric acid on estimated GFR in cardiac surgery patients: A pilot study. Am J Nephrol 2015;42:402–409. 10.1159/000443283 26731594

[24] Akbar SR , Long DM , Hussain K , Alhajhusain A , Ahmed US , Iqbal HI , *et al*. Hyperuricemia: An early marker for severity of illness in sepsis. Int J Nephrol 2015;2015:301021. 10.1155/2015/301021 26294973 PMC4532866

[25] Cheungpasitporn W , Thongprayoon C , Harrison AM , Erickson SB . Admission hyperuricemia increases the risk of acute kidney injury in hospitalized patients(.). Clin Kidney J 2016;9:51–56. 10.1093/ckj/sfv086 26798461 PMC4720187

[26] Greenberg KI , McAdams‐DeMarco MA , Köttgen A , Appel LJ , Coresh J , Grams ME . Plasma urate and risk of a hospital stay with AKI: The Atherosclerosis Risk in Communities study. Clin J Am Soc Nephrol 2015;10:776–783. 10.2215/CJN.05870614 25717072 PMC4422233

[27] Ohara K , Masuda T , Morinari M , Okada M , Miki A , Nakagawa S , *et al*. The extracellular volume status predicts body fluid response to SGLT2 inhibitor dapagliflozin in diabetic kidney disease. Diabetol Metab Syndr 2020;12:37. 10.1186/s13098-020-00545-z 32377235 PMC7195732

[28] van Bommel EJM , Muskiet MHA , van Baar MJB , Tonneijck L , Smits MM , Emanuel AL , *et al*. The renal hemodynamic effects of the SGLT2 inhibitor dapagliflozin are caused by post‐glomerular vasodilatation rather than pre‐glomerular vasoconstriction in metformin‐treated patients with type 2 diabetes in the randomized, double‐blind RED trial. Kidney Int 2020;97:202–212. 10.1016/j.kint.2019.09.013 31791665

[29] Heerspink HJL , Stefánsson BV , Correa‐Rotter R , Chertow GM , Greene T , Hou FF , *et al*.; DAPA‐CKD Trial Committees and Investigators . Dapagliflozin in patients with chronic kidney disease. N Engl J Med 2020;383:1436–1446. 10.1056/NEJMoa2024816 32970396

[30] McAdams DeMarco MA , Maynard JW , Baer AN , Gelber AC , Young JH , Alonso A , *et al*. Diuretic use, increased serum urate levels, and risk of incident gout in a population‐based study of adults with hypertension: The Atherosclerosis Risk in Communities cohort study. Arthritis Rheum 2012;64:121–129. 10.1002/art.33315 22031222 PMC3253199

