# Supplementary material for: Effects of empagliflozin on uric acid levels during acute heart failure recompensation: A sub‐analysis of the EMPAG‐HF trial (Effects of Empagliflozin on Diuresis and Renal Function in Patients with Acute Decompensated Heart Failure)
Source: Eur J Heart Fail. 2025 Jul 9;27(8):1472–8. doi: 10.1002/ejhf.3723 (PMC12482834; doi:10.1002/ejhf.3723)
Supplement: Supplementary file 1 — Appendix S1. Supporting Information. [file EJHF-27-1472-s001.docx]

Supplemental material


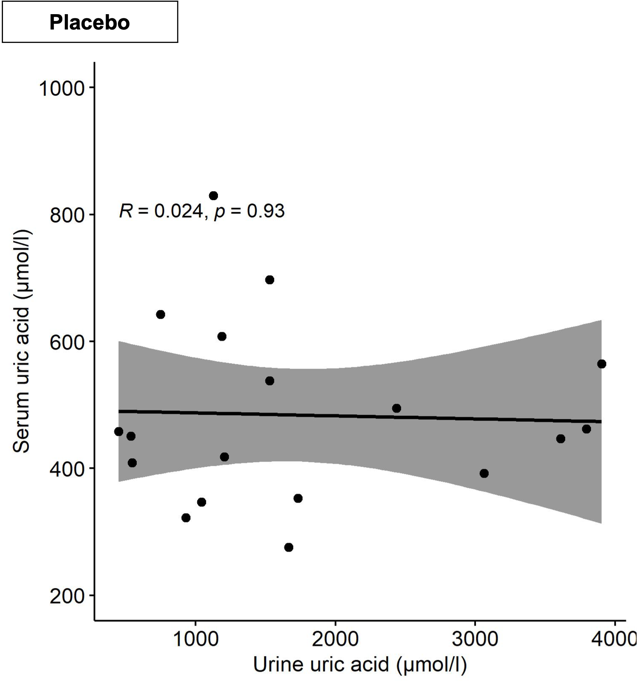

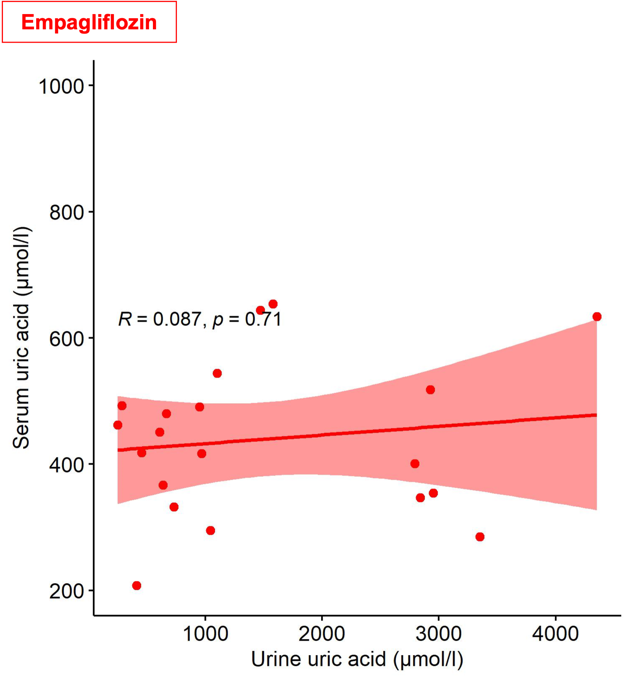


Figure 1: Correlation between serum and urine uric acid


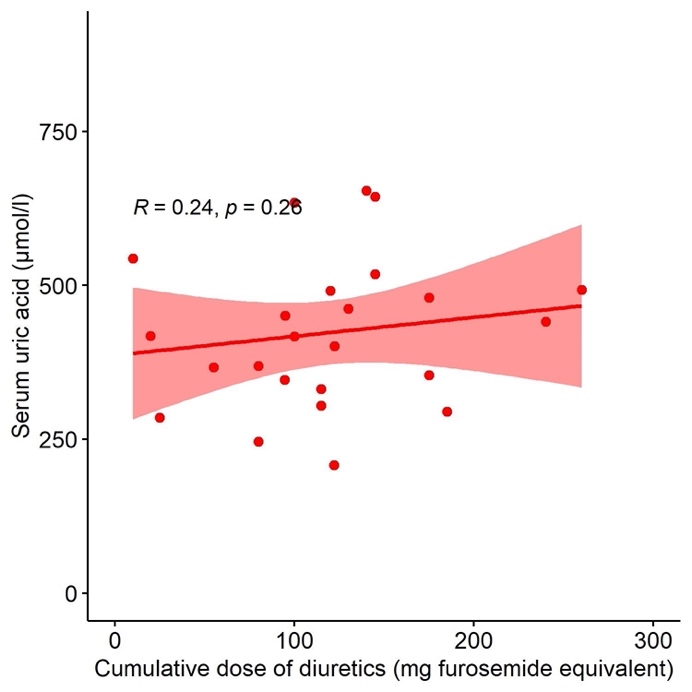


Figure 2: Correlation between serum uric acid levels and cumulative dose of diuretics in the treatment group


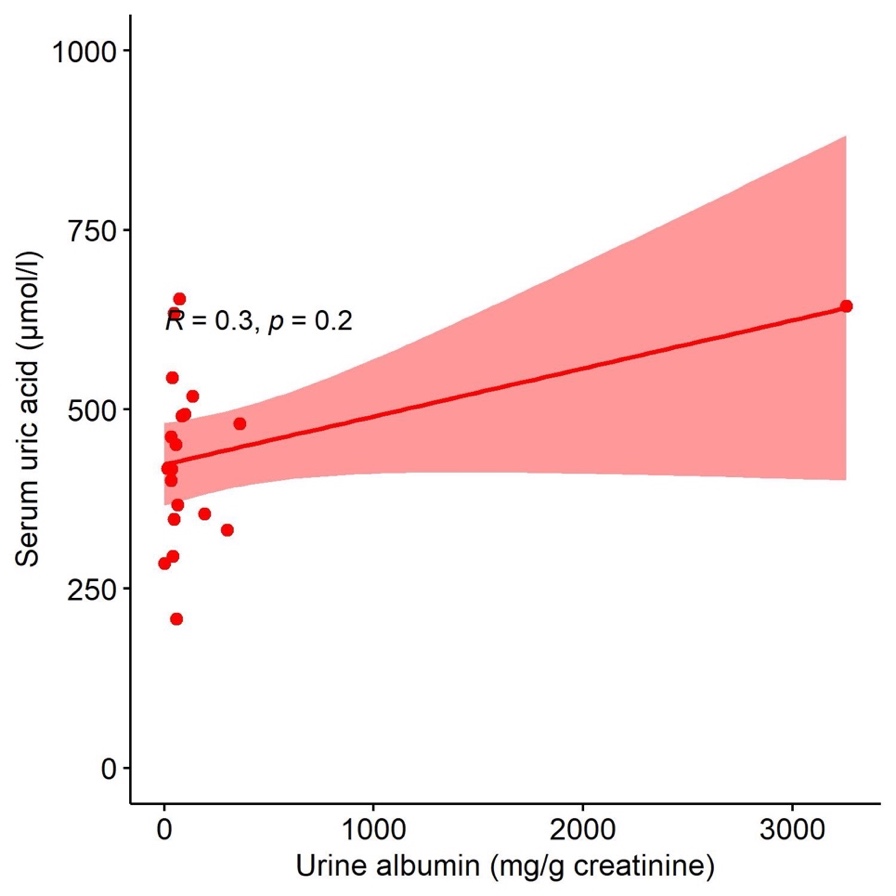


Figure 3: Correlation between urine albumin and serum uric acid on day 5


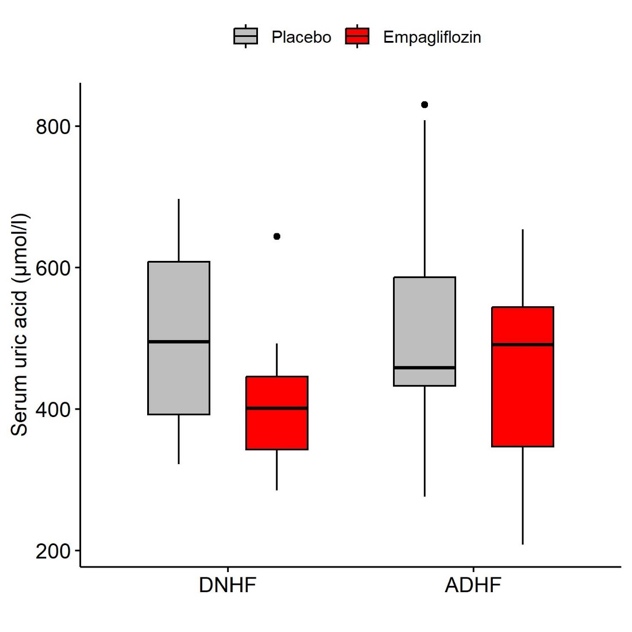

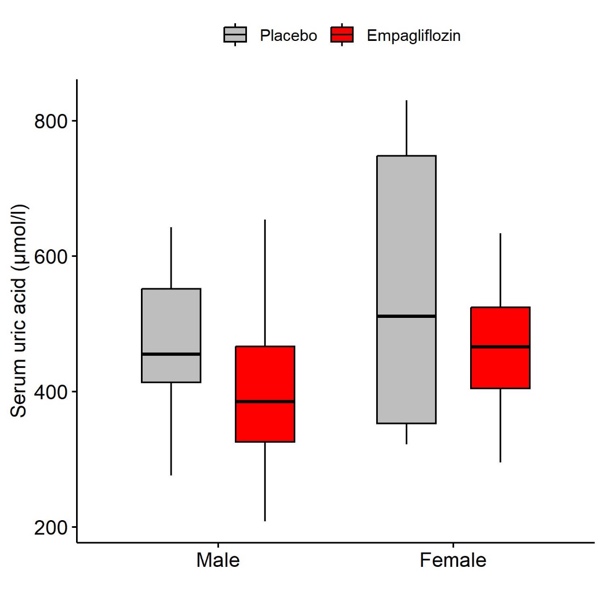


Figure 4: Serum uric acid in de novo heart failure and according to sex

**Diabetes**

|  | **Baseline** | | **Day 2** | **Day 3** | **Day 4** | **Day 5** | **Day 30** |
| --- | --- | --- | --- | --- | --- | --- | --- |
| **Placebo** | | 446.12 ± 35.53 | 467.25 ± 37.92 | 480.29 ± 52.05 | 494.00 ± 56.22 | 471.22 ± 53.05 | 424.33 ± 48.17 |
| **Empagliflozin** | | 480.36 ± 35.58 | 469.25 ± 34.33 | 453.82 ± 40.04 | 456.20 ± 42.59 | 449.90 ± 47.13 | 544.50 ± 44.22 |

**Non-diabetes**

|  | **Baseline** | **Day 2** | **Day 3** | **Day 4** | **Day 5** | **Day 30** |
| --- | --- | --- | --- | --- | --- | --- |
| **Placebo** | 511.71 ± 46.10 | 516.94 ± 38.22 | 527.33 ± 36.24 | 529.87 ± 38.37 | 531.07 ± 38.46 | 539.50 ± 43.25 |
| **Empagliflozin** | 439.15 ± 29.45 | 428.93 ± 26.44 | 421.08 ± 29.26 | 401.20 ± 28.25 **#** | 404.07 ± 26.30 **#** | 447.73 ± 47.23 |

Data are presented as mean ± SEM. #: significantly different vs. placebo at the same time points (Day 4: p = 0.012, Day 5: p = 0.012).

Table 1: Stratified analysis by the presence of diabetes
